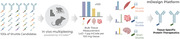# Rapid discovery and optimization of novel brain shuttles and shuttle‐enabled anti‐amyloid therapeutics using multiplexed in vivo biologics design

**DOI:** 10.1002/alz.095642

**Published:** 2025-01-09

**Authors:** Shane Lofgren

**Affiliations:** ^1^ Manifold Bio, Boston, MA USA

## Abstract

**Background:**

Drug delivery to the central nervous system has long been hindered by the restrictive properties of the blood‐brain barrier (BBB). Recent advances have highlighted the ability of certain antibody “shuttles'' to traverse the BBB and enhance the delivery of diverse therapeutic payloads. However the true potential of this approach remains underexplored and is limited by traditional *in vitro* screening platforms that fail to capture the complexities of *in vivo* biology. Novel technologies that facilitate the development of brain shuttles and shuttle‐enabled neuromedicines could improve the uptake, efficacy, safety, and dosing of broad classes of therapeutics, and transform the treatment of vast numbers of patients with neurological disease.

**Method:**

Here, we introduce a high‐throughput in vivo screening methodology based on Manifold’s mCodeTM technology to evaluate BBB penetration and uptake of over 1000 brain shuttling antibody candidates targeting 10+ different receptors (Figure).

**Result:**

Our approach reveals antibody shuttles with diverse molecular properties and distinct pharmacokinetic profiles in brain, blood, and other peripheral tissues. We leverage this large *in vivo* dataset using machine learning to further engineer these antibodies for enhanced brain uptake and residence time. Engineering of brain shuttles with anti‐amyloid antibodies against BACE1 and amyloid beta significantly improved the uptake and efficacy of these fusions versus unshuttled controls and comparator shuttled molecules.

**Conclusion:**

Collectively, our work (1) expands the pool of known targets and molecules capable of shuttling therapeutic payloads across the BBB, (2) validates the utility of these molecules toward the design of novel neuromedicines for Alzheimer’s disease, and (3) signifies a paradigm shift in utilizing thoughtfully‐designed, high‐throughput *in vivo* screening in early stages of drug discovery.